# Salivary Calculi

**Published:** 1898-02

**Authors:** A. S. Wolff

**Affiliations:** Brownsville, Texas


### Salivary Calculi.

*Editor Texas Medical Journal:*

During the last few days I have been looking over some of my old notes, and it occurred to me that some of the cases might be of interest to some of the readers of your valuable JOURNAL, I make the cases tell their own tales taken at the time the patients were under my own eyes.

I have no new facts extending the boundaries of the healing art, for the reason that now-a-days it is difficult to advance anything that has not already been previously related by some observer. I forward a salivary calculus, removed from Mr. F., a bookkeeper for one of our principal merchants here. F. at present resides at Laredo, Texas; originally he came from New Orleans. He informs me that he has been troubled on and off for years with a swelling in his mouth. On examination I found the whole buccal cavity in a high and active state of hyperæmia; the membrane of the palate swollen and livid; a large swelling of the angle of the right jaw; pain in the neck; fœtid discharge from the mouth. He informs me that he had the same trouble some four months previous, and while at New Orleans was attended by a doctor who, after giving him some medicine to wash his mouth something burst, and much discharge of pus came away; he soon got well; never suffered so much as he did at present. The want of sleep has exhausted him; cannot swallow food or water. The cause of it all is unintelligible to him, and so it was for the doctor. His protracted suffering impaired his usual good health; flesh, spirit, energy all gone.

On examination on both sides of the frœnum a tumor, involving the connective tissues, livid and boggy, full, occupying the mouth, pressing the tongue upwards and against the lower teeth. The tumor is fluctuating, as if it contained fluid. I advised immediate lancing, which he refused, giving as his reason for the non-use of the knife, that on several occasions he has had the same trouble; it always got better when it opened, and no doubt it would do so again. After reasoning with him for some time, he remained obdurate; he only wanted some medicine to hasten the thing to open. After he positively refused the use of the knife, I advised him to send for some one else. On my leaving the room he asked me to do something, if it was only to give him temporary relief. I then gave him a hypodermic injection of morphia and left. At home I was thinking about the case, and made up my mind that the trouble was some foreign body which at some time had found its way to the floor of the mouth. The next morning he sent for me again. On my arrival the poor fellow was groaning from pain, and willingly consented to allow me to make an incision; anything to give him relief.

After the usual disinfection of hands and instruments, I made a free incision at the most prominent part of the tumor, avoiding the ranine artery; an incroyable quantity of foul smelling pus gushed out, when he fainted; he came out of this very soon, but the discharge kept up for some time. When the pus stopped flowing the cavity was syringed out with warm water; I then passed my finger into the cavity, which came in contact with some hard substance; took hold of it with forceps, but could not bring it forward; the substance, whatever it was, was strongly adherent to the surrounding tissues. Passing a narrow blade of a small bistory along the forceps I easily separated the tissues above the foreign body. I then made out that I had to deal with calculi;—being so far denuded, I attempted to bring again forward, but the slightest traction produced great pain. The posterior and lateral adhesions were so firmly attached to the calculus, that I was obliged to dissect off the tissues. I then passed a small pair of curved scissors behind and slowly dissected out the large calculus I forward to you; large for the locality where it was found. The cavity was syringed and washed out with a solution of boracic acid, and filled with borated cotton. I forbear giving the trifling particulars of the subsequent treatment. But the man got well without further trouble.

The literature on the subject was fairly searched, but with scant benefit.

Among the many foreign bodies found in the economy, there are none so little considered in the text-books or medical literature as the salivary calculi. While it is admitted that the salivary calculi is not a common affection, and the fact of the paucity of literature on the subject, it may not be out of place to call attention to the subject.

A disease analogous to the salivary calculi is often mentioned in the books under the name of ranula, and is recognized as fluctuating tumor, and usually consists of a cyst, containing a glutinous mucous fluid, generally originating by occlusion of Wharton's duct, or may develop in any part of the buccal cavity, and which is disposed of by dissecting out the cyst, and evacuating its contents, while the salivary calculi generally originates in obstruction of the duct of the sublingual glands. Of course this case is of but small importance, but believing that the aggregate of cases from the personal experience of the country doctor, often of great interest, are lost for the want of record, which if noted would add to the common stock, but when confined to self, is lost to all.

The science of medicine consists of accumulated facts and experience of individuals. Every practitioner in his daily work often has cases giving anxiety and trouble. Many interesting facts escape consideration because no special indication are thought worthy of record and of value. This is a great mistake—much of interest and instruction is lost by this neglect to the common stock.

A. S. WOLFF, M. D.,
Brownsville, Texas.

